# Factors associated with smoking and risky drinking among university students in Japan

**DOI:** 10.1265/ehpm.26-00017

**Published:** 2026-05-09

**Authors:** Yuichiro Otsuka, Aya Kinjo, Hideyuki Kanda, Hisashi Yoshimoto, Teruna Ito, Yuki Kuwabara, Hongja Kim, Aya Iwamoto, Yoneatsu Osaki, Tomomi Miyoshi, Yuki Tanaka, Yoshitaka Kaneita

**Affiliations:** 1 Division of Public Health, Department of Social Medicine, Nihon University School of Medicine, 1-6 Kanda-Surugadai, Chiyoda-ku, Tokyo 101-8309, Japan; 2 Division of Environmental and Preventive Medicine, Department of Social Medicine, Faculty of Medicine, Tottori University, Yonago-city, Tottori 683-8503, Japan; 3 Department of Public Health, Okayama University Graduate School of Medicine, Dentistry and Pharmaceutical Sciences, Okayama-city, Okayama 700-8558, Japan; 4 Department of Family Medicine, General Practice and Community Health, Faculty of Medicine, University of Tsukuba, Tsukuba-city, Ibaraki 305-8576, Japan; 5 Department of Food and Nutrition, Koriyama Women’s University, Koriyama-city, Fukushima 963-8503, Japan

**Keywords:** Smoking, Risky drinking, University students, Sex differences, Japan

## Abstract

**Background:**

Little is known about smoking and alcohol consumption after high school in Japan. This study examined factors associated with smoking and risky drinking among Japanese university students, focusing on sex-related differences.

**Methods:**

A cross-sectional web-based survey was conducted among students from five Japanese universities. A total of 253 men and 510 women anonymously completed questionnaires assessing smoking, alcohol use, substance-related behaviors, and depressive symptoms. Logistic regression using penalized regression methods were performed to determine factors associated with smoking and risky drinking.

**Results:**

Overall, 7.7% students reported smoking and 25.6% were classified as risky drinkers. Peer smoking was strongly associated with current smoking in men (odds ratio [OR]: 4.58) and women (OR: 2.45). Family smoking and depressive symptoms were associated with smoking only among women, indicating a sex-specific pattern. Regarding risky drinking, common associated factors in both sexes included alcohol-related negative outcomes (OR: 5.83 for men, 5.49 for women) and current smoking (OR: 3.83 for men, 4.86 for women). Among men, additional factors associated with risky drinking were living alone (OR: 2.96) and mixing alcohol with energy drinks (OR: 4.53).

**Conclusions:**

After the age of 20, the rates of smoking and risky drinking might increase sharply among Japanese young adults. Sex-specific patterns indicate that male behaviors are shaped by external invitations and living situations, whereas female behaviors are linked to familial and psychological factors.

## Background

Smoking and alcohol consumption (hereafter drinking) commonly increase during the transition from adolescence to young adulthood, a developmental period marked by increased autonomy, social experimentation, and reduced parental supervision [1, 2]. Recent evidence suggests that the initiation of regular smoking has shifted toward early adulthood, and drinking typically rises after joining university, often peaking during the early 20s [2–4]. These behavioral changes contribute substantially to short- and long-term health risks, including injuries, mental health problems, and future non-communicable diseases [5, 6].

Among university students, several factors have been associated with smoking and risky drinking, including peer and family substance use, psychological distress, early initiation of smoking or alcohol use, and engagement in other risky behaviors [7–10]. Co-occurrence of smoking and heavy drinking is particularly common in this age group [11, 12], and emerging risk patterns such as alcohol mixed with energy drinks (AmED) have garnered increasing attention [13]. Sex-specific differences have been reported, with male students often engaging in higher levels of substance use, whereas psychological distress may play a more prominent role among female students [14, 15].

In Japan, smoking and drinking among high school students have declined to historically low levels [16]. In 2021, the smoking rates for grade 12 students were 1.0% for males and 0.8% for females, and the corresponding monthly drinking rates were 4.3% and 2.9% [17]. However, the 2023 National Health and Nutrition Survey of Japan showed that the smoking prevalence rate among individuals in their 20s was 13.1% (males: 20.5%, females: 5.2%), and the corresponding risky drinking rate was 6.0% (males: 4.3%, females: 7.8%) [18]. This transition suggests that young adulthood represents a critical stage for the adoption of smoking and risky drinking. In Japan, the legal age for drinking and smoking is 20 years, so although the increase in this prevalence suggests a possible influence, it has not been clearly established. Despite this pattern, research focusing specifically on university students in Japan remains limited. In a longitudinal study of university students conducted around the year 2000, risk factors associated with the initiation of smoking included male sex, skipping breakfast, frequently eating dinner out, habitual drinking, short sleep duration, and depression [19]. Regarding alcohol consumption, male students reported engaging in binge drinking primarily to experience happiness and alleviate stress. In contrast, female students indicated that their motivations for binge drinking included achieving happiness and stress reduction and forming social connections and escaping negative experiences [20]. These studies are quite dated or focus only on individuals aged 20 years and over, thereby failing to investigate the period between high school graduation and the age of 20 years.

Given these gaps, a clearer understanding of the factors associated with smoking and risky drinking among Japanese university students is needed to inform targeted prevention strategies. Therefore, we aimed to investigate the behavioral, psychological, and social correlations of smoking and risky drinking among university students in Japan, with particular attention paid to potential sex differences.

## Methods

### Participants and procedures

Study participants were recruited from five universities in the Tohoku, Kanto, and Chugoku regions of Japan, located in metropolitan or regional areas. Four universities were comprehensive, one accepted only economics students, and one was exclusively for women. Inclusion criteria were aged 18 years and enrollment in an undergraduate program; graduate students and those under 17 years were excluded. Invitations questionnaires were distributed via e-mail, and the web survey URL was posted on university bulletin boards. Participation was voluntary, anonymous, and withdraw was permitted at any time without penalty. Data were collected through the web survey platform. This study was conducted between December 2024 and June 2025 and approved by the Ethics Committee of Nihon University School of Medicine (approval number: 2024-14).

### Measures

The questionnaire collected information on demographic characteristics (age and sex), tobacco, alcohol consumption, drug use among acquaintances, experiences of being persuaded to use drugs, and mental health status (Patient Health Questionnaire-2 [PHQ-2]).

### Tobacco-related questions

The survey assessed (1) smoking status of various tobacco products, (2) age at smoking initiation, (3) current smoking status of family members, and (4) current smoking status of friends. Tobacco products were classified as conventional cigarettes, heated tobacco products (HTPs), e-cigarettes, smokeless tobacco, and waterpipes. Each product was described in the survey, with examples of popular brands provided (e.g., IQOS, Glo, Ploom Tech, Pulze, for HTPs; myblu, DR. VAPE, eGO AIO, ZERO STICK, VAPORESSO OSMALL for e-cigarettes; SNUS, VELO for smokeless tobacco; hookah, shisha for waterpipes). Current smokers were defined as participants reporting use of any of these products on at least one day in the previous 30 days.

### Alcohol consumption status

The survey assessed (1) risky drinking status, (2) age at drinking initiation, (3) experiences of drinking-related failure, (4) experiences of alcohol harassment, and (5) mixing alcohol with energy drinks. Alcohol consumption was evaluated using the three-item Alcohol Use Disorders Identification Test - Consumption (AUDIT-C), a World Health Organization screening tool [21], reflecting respondents’ drinking habits over the past year. Risky drinkers were defined an AUDIT-C score ≥4 for men and ≥3 for women, based on previous validation studies [21, 22]. Alcohol harassment included verbal, physical, or psychological abuse; sexual harassment; or being forced to drink. Alcohol-related failures were defined as incidents within the past year resulting from drinking, such as vomiting, fights, memory loss, police apprehension, reprimands by parents or teachers, or injury. Participants reporting mixing alcohol with energy drinks at least once per year were classified as having experience with AmED.

### Covariates

Regarding substance use, the following two questions were posed: (1) “In the past year, has anyone close to you overdosed on prescription medication or used marijuana, inhalants (thinners), or dangerous drugs?” and (2) “Have you ever overdosed on prescription medication or used marijuana, inhalants, or dangerous drugs?” Participants answering “yes” to either question were considered affirmative.

Mental health was evaluated using the PHQ-2, comprising the first two items from the PHQ-9 [23]. The PHQ-2 is a validated screening instrument for depression. Participants reported how often over the past two weeks they had (1) little interest or pleasure in activities and (2) felt down, depressed, or hopeless over the previous 2 weeks, by using a scale ranging from 0 (not at all) to 3 (nearly every day). Total scores range from 0 to 6, with a cutoff of 3 indicating potential risk for depression [24, 25].

### Statistical analyses

Participants’ demographics and the prevalence of smoking and alcohol consumption were first examined, with results stratified by sex. Categorical variables are presented as proportions with 95% confidence intervals (CIs). To explore factors associated with smoking, univariate logistic regression (Model 1) was performed, including age, sex, living status, family and peer smoking, smoking initiation at ≤17 years, risky drinking, peers’ substance use or invitations, and depression as explanatory variables. Variables with p < 0.15 in the univariate analysis were included in multivariable logistic regression (Model 2) [26, 27]. The variable “smoking initiation at ≤17 years” was excluded from the multivariable model due to the small sample size.

Similarly, factors associated with risky drinking were examined using univariate logistic regression (Model 1), including age, sex, living status, drinking-related failures, alcohol harassment, mixing alcohol with energy drinks, drinking initiation at ≤17 years, current smoking, peers’ substance use or invitations, and depression. Variables with p < 0.15 in the univariate analysis were entered into the multivariable model (Model 2) [26, 27]. Lastly, as a sensitivity analysis, we repeated the same. Sensitivity analyses were conducted stratified by sex. All logistic regressions used Firth- and logF-type penalized methods for small or sparse data sets [28]. Multicollinearity was assessed via variance inflation factors, all <5, indicating no significant collinearity. Statistical significance was set at p < 0.05. Analyses were performed using Stata version 18.0 (Stata Corp).

## Results

### Participants

Table 1 shows the participants’ demographic characteristics. Overall, 763 students (253 men, 510 women) participated in this study. The participants were predominantly young adults, with 85.0% aged 18–21 years. The most prevalent health-risk behavior was risky drinking, identified in 25.6% of the participants. Risky drinking was more common in women (28.0%) than in men (20.6%). Furthermore, 20.3% of the participants reported experiencing drinking-related failure, and 27.7% had experienced alcohol harassment. Other substance-use behaviors were rare; only 1.2% had started smoking at the age of ≤17 years, and peer substance use was reported by 3.7%. The prevalence of depression in the sample was 12.7%.

**Table 1:** Demographics of the survey participants

	**Total (N = 763)**		**Men (n = 253)**		**Women (n = 510)**	

	**n**	**%**	**n**	**%**	**n**	**%**
Age						
18–19 years	368	48.2	138	54.5	230	45.1
20–21 years	281	36.8	76	30.0	205	40.2
over 22 years	114	14.9	39	15.4	75	14.7
Living type						
Single	357	46.8	140	55.3	217	42.5
Smoking in the family						
Yes	297	38.9	76	30.0	221	43.3
Smoking in the peers						
Yes	167	21.9	60	23.7	107	21.0
Start smoking at age ≤17 years						
Yes	9	1.2	4	1.6	5	1.0
Risky drinking						
Yes	195	25.6	52	20.6	143	28.0
Start drinking at age ≤17 years						
Yes	28	3.7	13	5.1	15	2.9
Experience of failure due to drinking						
Yes	155	20.3	48	19.0	107	21.0
Experience of received alcohol harassment						
Yes	211	27.7	67	26.5	144	28.2
Experience of mixed energy drink and alcohol drinking						
Yes	60	7.9	22	8.7	38	7.5
Substance use behavior of peers or Invitation to substance use behavior						
Yes	37	4.9	16	6.3	21	4.1
Depression						
Yes	97	12.7	31	12.3	66	12.9

Risky drinking was defined as an AUDIT-C score ≥4 points in men and ≥3 points in women. AUDIT-C: Alcohol Use Disorders Identification Test - Consumption

### Smoking and drinking statuses

Table 2 shows the prevalence of smoking and risky drinking among university students according to sex. The prevalence rates of total smoking and the use of conventional cigarettes, HTPs, e-cigarettes, smokeless tobacco, and water pipes were 7.7% (95% CI: 5.9–9.9%), 5.2% (95% CI: 3.8–7.1%), 1.3% (95% CI: 0.6–2.4%), 0.3% (95% CI: 0.0–0.9%), and 1.2% (95% CI: 0.5–2.2%), respectively. Among the male participants, the prevalence of conventional cigarette use was the highest (10.7%; 95% CI 7.2–15.1%). Conversely, among the female participants, HTPs use was the most prevalent (3.3%; 95% CI 2.0–5.3%). Notably, no male participant reported smokeless tobacco use. The total prevalence of risky drinking was 25.6% (20.6% and 28.0% in male and female participants, respectively).

**Table 2:** Prevalence of smoking and alcohol drinking among participants by sex

	**Total (N = 763)**			**Men (n = 253)**			**Women (n = 510)**		

	**n**	**%**	**95% CI**	**n**	**%**	**95% CI**	**n**	**%**	**95% CI**
Smoking	59	7.7	5.9–9.9	29	11.5	7.8–16.0	30	5.9	4.0–8.3
Conventional cigarettes	40	5.2	3.8–7.1	27	10.7	7.2–15.1	13	2.5	1.4–4.3
HTPs	26	3.4	2.2–5.0	9	3.6	1.6–6.6	17	3.3	2.0–5.3
E-cigarettes	10	1.3	0.6–2.4	4	1.6	0.4–4.0	6	1.2	0.4–2.5
Smokeless tobacco	2	0.3	0.0–0.9	0	0.0		2	0.4	0.0–1.4
Water pipes	9	1.2	0.5–2.2	1	0.4	0.0–2.2	8	1.6	0.7–3.1
Risky drinking	195	25.6	22.5–28.8	52	20.6	15.7–26.1	143	28.0	24.2–32.2

Risky drinking was defined as an AUDIT-C score ≥4 points in men and ≥3 points in women. AUDIT-C: Alcohol Use Disorders Identification Test – Consumption; HTP: heated tobacco product; CI: confidence interval.

### Factors associated with smoking among university students

Table 3 shows the factors associated with smoking among university students in Japan. Multivariable logistic regression analysis showed that higher age, smoking among peers (odds ratio [OR]: 2.87, 95% CI: 1.55–5.33), risky drinking (OR: 7.01, 95% CI: 3.35–14.64), and substance use behavior of peers or invitation to substance use behavior (OR: 2.65, 95% CI: 1.05–6.67) were significantly associated with smoking. Among men, smoking among peers (OR: 4.58, 95% CI: 1.62–12.95) and risky drinking (OR: 8.76, 95% CI: 2.97–25.83) were significantly associated with smoking. Among women, in addition to these factors, smoking in the family (OR: 2.67, 95% CI: 1.11–6.42) and depression (OR: 3.26, 95% CI: 1.37–7.76) were also significantly associated with smoking.

**Table 3:** Prevalence of smoking and factors associated with among university students

	**Total (N = 763)**					**Men (n = 253)**					**Women (n = 510)**				

		**Model 1**		**Model 2**			**Model 1**		**Model 2**			**Model 1**		**Model 2**	

	**%**	**OR**	**95% CI**	**OR**	**95% CI**	**%**	**OR**	**95% CI**	**OR**	**95% CI**	**%**	**OR**	**95% CI**	**OR**	**95% CI**
Sex															
Male	11.5	1.00													
Female	5.9	0.73	0.18–2.45												
Age															
18–19 years	1.4	0.14**	0.18–2.45	0.42	0.15–1.20	2.2	0.17**	0.05–0.65	0.37	0.09–1.59	0.9	0.11**	0.02–0.48	0.51	0.12–2.20
20–21 years	11.7	1.00		1.00		18.4	1.00		1.00		9.3	1.00		1.00	
>22 years	18.4	2.29**	1.23–9.28	2.06*	1.03–4.11	30.8	2.00*	1.23–9.28	2.88	0.88–9.39	12.0	1.58	0.67–3.74	1.24	0.49–3.17
Living															
Not single	9.4	1.00				15.0	1.00				7.2	1.00			
Single	5.9	0.80	0.45–1.42			8.6	1.15	0.48–2.74			4.2	0.62	0.28–1.39		
Smoking in the family															
No	5.8	1.00		1.00		10.7	1.00				2.8	1.00		1.00	
Yes	10.8	2.16**	1.24–3.77	1.46	0.78–2.73	13.2	1.10	0.47–2.60			10.0	4.14**	1.78–9.61	2.67*	1.11–6.42
Smoking in the peers															
No	4.2	1.00		1.00		6.2	1.00		1.00		3.2	1.00		1.00	
Yes	20.4	4.85**	2.75–8.57	2.87**	1.55–5.33	28.3	4.59**	1.95–10.85	4.58**	1.62–12.95	15.9	5.10**	2.37–10.97	2.45*	1.07–5.60
Start smoking at age ≤17 years															
No	7.0	1.00				10.0					5.5	1.00			
Yes	66.7	22.67**	4.84–106.08			100					40.0	12.82**	1.95–84.20		
Risky drinking															
No	1.9	1.00		1.00		3.5	1.00		1.00		1.1	1.00		1.00	
Yes	24.6	13.93**	6.98–27.8	7.01**	3.35–14.64	42.3	16.57**	6.23–43.91	8.76**	2.97–25.83	18.2	17.86**	6.04–52.80	7.46**	2.50–22.23
Substance use behavior of peers and/or Invitation to substance use behavior															
No	6.8	1.00		1.00		9.3	1.00		1.00		5.5	1.00		1.00	
Yes	27.0	3.65**	1.60–8.33	2.65*	1.05–6.67	43.8	4.11**	1.30–12.98	2.77	0.70–10.96	14.3	2.36	0.96–5.79	1.89	0.51–7.00
Depression															
No	6.6	1.00		1.00		11.7	1.00				4.1	1.00		1.00	
Yes	15.5	2.25*	1.17–4.34	1.72	0.82–3.61	9.7	0.67	0.18–2.45			18.2	4.61**	1.37–8.26	3.26**	1.37–7.76

Model 1 was univariate logistic regression analysis only adjusted for universities

Model 2 was multivariable logistic regression analysis using Firth- and logF-type penalized regression methods adjusted above variables and universities.

Risky drinking was defined as an AUDIT-C score of ≥4 points in men and ≥3 points in women. AUDIT-C: Alcohol Use Disorders Identification Test – Consumption; CI: confidence interval; OR: odds ratio. *P < 0.05; **P < 0.01.

### Factors associated with risky drinking among university students

Table 4 shows the factors associated with risky drinking among university students in Japan. Multivariable logistic regression analysis showed that female sex (OR: 1.86, 95% CI: 1.13–3.04), age, experience of drinking-related failure (OR: 5.65, 95% CI: 3.52–9.07), having AmED (OR: 2.28, 95% CI: 1.10–4.75), and present smoking (OR: 4.62, 95% CI: 2.07–10.32) were significantly associated with risky drinking. Among men, in addition to the same explanatory variables, single living (OR: 2.96, 95% CI: 1.14–7.72) was significantly associated with risky drinking. In contrast, among women, lower age (OR: 0.11, 95% CI: 0.05–0.24) experience of drinking-related failure (OR: 5.65, 95% CI: 3.52–9.07), and present smoking (OR: 4.86, 95% CI: 1.59–14.87) were significantly associated with risky drinking.

**Table 4:** Prevalence and factors associated with risky drinking among university students in Japan

	**Total (N = 763)**					**Men (n = 253)**						**Women (n = 510)**			

		**Model 1**		**Model 2**			**Model 1**		**Model 2**			**Model 1**		**Model 2**	

	**%**	**OR**	**95% CI**	**OR**	**95% CI**	**%**	**OR**	**95% CI**	**OR**	**95% CI**	**%**	**OR**	**95% CI**	**OR**	**95% CI**
Sex															
Male	20.6	1.00		1.00											
Female	28.0	1.89**	1.28–2.78	1.86*	1.13–3.04										
Age															
18–19 years	4.1	0.06**	0.03–0.11	0.13**	0.07–0.24	3.6	0.09**	0.03–0.26	0.22**	0.07–0.65	4.4	0.06**	0.03–0.11	0.11**	0.05–0.24
20–21 years	44.1	1.00		1.00		32.9	1.00		1.00		48.3	1.00		1.00	
>22 years	58.0	1.34	0.86–2.10	1.36	0.80–2.31	56.4	3.13*	1.36–7.18	3.28*	1.20–8.95	45.3	1.00	0.58–1.73	0.96	0.51–1.78
Living															
Not single	28.6	1.00				18.6	1.00		1.00		32.4	1.00		1.00	
Single	22.1	0.83	0.59–1.16			22.1	1.95	0.98–3.89	2.96*	1.14–7.72	22.1	0.65*	0.43–0.98	0.78	0.47–1.31
Experience of failure due to drinking (ref: No)															
No	13.8	1.00		1.00		9.8	1.00		1.00		15.9	1.00		1.00	
Yes	71.6	15.07**	9.87–23.03	5.65**	3.52–9.07	66.7	17.63**	8.16–38.13	5.83**	2.37–14.36	73.8	14.22**	8.49–23.82	5.49**	3.14–9.61
Experience of received alcohol harassment															
No	23.7	1.00		1.00		21.0	1.00				25.1	1.00		1.00	
Yes	30.3	1.33	0.93–1.91	0.78	0.49–1.25	19.4	0.79	0.38–1.62			35.4	1.62*	1.06–2.48	0.99	0.57–1.70
Experience of mixed energy drink and alcohol drinking (ref: No)															
No	22.1	1.00		1.00		16.0	1.00		1.00		25.0	1.00		1.00	
Yes	66.7	6.06**	3.41–10.77	2.28*	1.10–4.75	68.2	8.83**	3.26–23.86	4.53*	1.19–17.25	65.8	5.42**	2.65–11.07	1.94	0.79–4.78
Start drinking at age ≤17 years (ref: No)															
No	25.0	1.00		1.00		20.0	1.00				27.5	1.00		1.00	
Yes	39.3	1.73	0.78–3.83	0.84	0.32–2.23	30.8	1.46	0.42–5.07			46.7	2.38	0.83–6.87	0.72	0.20–2.55
Present smoking															
No	20.9	1.00		1.00		13.4	1.00		1.00		24.4	1.00		1.00	
Yes	81.4	13.65**	6.84–27.24	4.62**	2.07–10.32	75.9	4.71*	1.24–17.95	3.83*	1.13–13.04	86.7	17.59**	5.96–51.91	4.86**	1.59–14.87
Substance use behavior of peers and/or Invitation to substance use behavior															
No	24.4	1.00		1.00		18.6	1.00		1.00		27.2	1.00		1.00	
Yes	48.7	2.44*	1.23–4.83	1.85	0.76–4.52	50.0	3.23*	1.11–9.39	4.08	0.96–17.25	47.6	2.36	0.96–5.78	1.48	0.48–4.56
Depression															
No	24.2	1.00		1.00		20.7	1.00				25.9	1.00		1.00	
Yes	35.1	1.55	0.97–2.45	1.20	0.67–2.15	19.4	0.85	0.32–2.22			42.4	1.84*	1.06–3.17	1.17	0.59–2.34

Model 1 was univariate logistic regression analysis only adjusted for universities

Model 2 was multivariable logistic regression analysis using Firth- and logF-type penalized regression methods adjusted above variables and universities.

Risky drinking was defined as an AUDIT-C score of ≥4 points in men and ≥3 points in women. AUDIT-C: Alcohol Use Disorders Identification Test – Consumption; CI: confidence interval; OR: odds ratio. *P < 0.05; **P < 0.01.

## Discussion

To our knowledge, this is the first study to comprehensively assess the factors associated with smoking and risky drinking among young adults in Japan. The main findings were as follows. First, the rates of smoking and risky drinking increased sharply after the age of 20 years. Second, smoking in the peers and engaging in risky drinking were common factors for smoking in both men and women. Additionally, smoking in the family and depressive symptoms were related factors for women. Finally, lower age, experiences of drinking-related failure, and current smoking were common positively related factors for risky drinking in both men and women. For men, living alone and experiences of AmED were also related factors. Substance use-related behaviors in one’s surroundings were not associated with smoking and risky drinking.

In this study, both smoking and hazardous drinking were significantly more prevalent among students aged 20 years and older. Similarly, a 2019 nationwide survey of individuals aged 18–24 years residing in major urban areas in Japan showed that the smoking prevalence was higher in those aged 20–24 years than in those aged 18–19 years, reflecting the legal age for smoking in Japan, and was notably higher in men than in women [29]. The 2021 National Survey of Adolescents found that the monthly alcohol consumption rate among high school students was 2.8%, whereas the 2023 National Health and Nutrition Survey reported 41.8% among people in their 20s. These findings suggest that age restrictions contribute to reduced smoking and drinking among minors, although respondents under 20 years may have consciously underreported their smoking and drinking behaviors.

In Japan, the full enforcement of the revised Health Promotion Act in April 2020 largely prohibited smoking in indoor public places, including restaurants and bars. Although this regulation reduced indoor smoking in bars and pubs, its effect on restaurants and cafes was limited [30], suggesting that smoking rates may not have substantially declined among the study population. Participants in this study experienced the COVID-19 pandemic during adolescence or upon entering university, which may also have influenced their drinking and smoking behaviors. Previous studies have reported that alcohol consumption among young people was suppressed [31], whereas smoking patterns remained largely stable [32]. Regional differences in COVID-19 incidence may have further affected drinking behaviors [33]. International evidence shows that post-pandemic binge drinking increased, while tobacco consumption remained stable among younger Spanish adolescents [34]. Future studies should investigate the impact of COVID-19 on youth smoking and drinking using longitudinal or repeated cross-sectional designs.

Our results showed a strong association between smoking and risky drinking, consistent with previous studies. Several factors may explain this link. Social settings, such as parties, facilitate concurrent alcohol and tobacco use through availability and peer influence [35, 36]. Smoking while drinking serves social functions, including acceptability, structuring time at events, and stress management [35]. Alcohol and nicotine act synergistically on reward pathways, reinforcing co-use and increasing the risk of alcohol disorders [37]. Male gender and higher physical activity correlate with increased rates of both behaviors in college populations [38].

Previous studies reported that the factors associated with smoking among university students include male sex, alcohol consumption, having family members or close friends who smoke, lower education level, psychological distress, and engagement in other high-risk behaviors (such as drug abuse and extramarital sex) [9, 39, 40]. Thus, the findings of our study are largely in agreement with the previous results.

Our study identified sex-based differences in factors related to smoking, with associations with familial smoking and depressive symptoms observed only among women. Longitudinal and meta-analytic studies demonstrate that youth are significantly more likely to initiate smoking if their parents or close peers smoke [41]. The impact of role modeling by parents is more pronounced in older adolescents, whereas peer influence is dominant in later adolescence [42]. A study of Saudi Arabian college students showed male students imitated their fathers, while females imitated their sisters, also revealing a significant correlation between their and friends’ smoking habits [43]. Differences from other studies may be due to cultural and religious factors. In any case, the findings suggest that smoking is easily influenced by those close to oneself, indicating that having appropriate role models throughout life may be an effective countermeasure [44]. Similar to our finding, a cross-sectional study of 1,214 undergraduate introductory psychology students showed that women with a history of depression were more likely to be smokers [45]. The possible underlying factors are as follows: (1) women are more prone to depression, and (2) women may be more likely than men to use smoking as a way to cope with stress.

In line with this study, several studies have reported that factors associated with risky drinking among university students include an early age of alcohol use onset, positive alcohol expectancies, living away from the family home, smoking, peer alcohol consumption, and polydrug use [46–48]. Additionally, a systematic review showed that the consumption of AmED was associated with risky alcohol drinking and alcohol dependence among university students [49]. The AmED alters neurotransmission involving adenosine and dopamine. Alcohol increases adenosine activity, causing sedation, while caffeine blocks adenosine receptors, counteracting sedation and boosting stimulation [50]. This blockade also enhances dopamine release, intensifying alcohol’s rewarding effects. As a result, the AmED may lead to greater drinking and a higher risk of dependence compared to that associated with alcohol alone [50].

Consistent with previous research [51, 52], we found experiences of negative drinking consequences were positively linked to risky drinking. Cognitive, behavioral, and neurobiological processes are proposed for these associations. Alcohol expectancy theory suggests individuals form beliefs about alcohol-related outcomes based on experiences. Negative consequences can paradoxically increase perceived likelihood and positivity of those consequences for some, reinforcing risky drinking. This process varies across individuals and consequence types, indicating some drinkers continue despite adverse experiences [53]. In young adulthood, they increase negative expectancies but not necessarily reduce alcohol use [54]. Neurobiological mechanisms show repeated negative consequences drive habit formation through changes in brain circuitry, particularly the basal ganglia and extended amygdala. Withdrawal states can motivate further drinking to alleviate discomfort [55]. Additionally, individuals with higher alcohol demand report more negative consequences, but their evaluation often does not deter future drinking [56].

This study has some limitations. First, this study recruited participants using university bulletin boards, which may have introduced selection bias, as the sample may overrepresent students with a greater interest in health-related issues. Although the multivariate analysis was adjusted for university, one of the participating institutions was a women’s university, resulting in a higher proportion of female respondents than male respondents, which may limit the generalizability of the findings. Second, the cross-sectional design precludes the establishment of causal relationships. Third, since the legal age for smoking and drinking in Japan is 20 years and data on these behaviors were self-reported, respondents might have underreported their smoking and drinking behaviors. Using a self-administered questionnaire introduces the potential for bias. Additionally, information regarding the frequency and quantity of alcohol consumption over the past year could be affected by recall bias. Consequently, the associations of smoking and alcohol consumption might have been somewhat underestimated.

Despite these limitations, our study has several strengths. These include the comprehensive nature of the tobacco and alcohol survey conducted among young adults and the application of well-validated tools to assess smoking, alcohol consumption, and mental health.

In conclusion, this study examined factors associated with smoking and drinking among university students, with analyses stratified by sex. While several factors were common to both sexes, notable differences were also observed, highlighting the need for both universal and sex-specific interventions. Routine screening for smoking and alcohol consumption during university health checkups can identify students at risk, who may benefit from brief preventive counseling or more intensive interventions. Future research should continue to monitor smoking and drinking behaviors during the post-high school transition and investigate longitudinally whether these factors identified in this study predict future risk.

## Abbreviations

AmED: alcohol mixed with energy drinks
AUDIT-C: Alcohol Use Disorders Identification Test – Consumption
CI: confidence interval
HTPs: heated tobacco products
OR: odds ratio
PHQ-2: Patient Health Questionnaire-2
VIF: variance inflation factor

## Declarations

### Availability of data and material

The data that support the findings of this study are available on request from the corresponding author. The data are not publicly available due to ethical restrictions.
